# Historical Feature Pattern Extraction Based Network Attack Situation Sensing Algorithm

**DOI:** 10.1155/2014/473504

**Published:** 2014-04-27

**Authors:** Yong Zeng, Dacheng Liu, Zhou Lei

**Affiliations:** ^1^Department of Industrial Engineering, Tsinghua University, Beijing 100084, China; ^2^Bengbu Automobile NCO Academy, Bengbu 233011, China; ^3^Tianjin Port (Group), Ltd., Tianjin 300456, China

## Abstract

The situation sequence contains a series of complicated and multivariate random trends, which are very sudden, uncertain, and difficult to recognize and describe its principle by traditional algorithms. To solve the above questions, estimating parameters of super long situation sequence is essential, but very difficult, so this paper proposes a situation prediction method based on historical feature pattern extraction (HFPE). First, HFPE algorithm seeks similar indications from the history situation sequence recorded and weighs the link intensity between occurred indication and subsequent effect. Then it calculates the probability that a certain effect reappears according to the current indication and makes a prediction after weighting. Meanwhile, HFPE method gives an evolution algorithm to derive the prediction deviation from the views of pattern and accuracy. This algorithm can continuously promote the adaptability of HFPE through gradual fine-tuning. The method preserves the rules in sequence at its best, does not need data preprocessing, and can track and adapt to the variation of situation sequence continuously.

## 1. Introduction

With attacks becoming more prevalent, the traditional static passive defense and whole system consolidation are hard to keep up with the changing rhythms, which have huge amounts of investment and affect the network performance. In this case, the dynamic, proactive, and targeted defending measures have been presented, most of which rely on attack situation forecast, that is, network attack situation sensing (NASS) [1, 2]. NASS aims at forecasting future evolution trend of network attack situation based on historical features and current attack indications, guiding dynamic defense, and allowing administrators to take corresponding measures in advanced, and effective manner to quickly respond to the complex and ever-changing attack threats [3, 4].

Rarely studying attack situation forecast, previous researches mostly using existing methods, such as autoregressive moving average model (ARMA), grey model (GM), and radial basis function neural network (RBFNN) [5–8]. ARMA identifies the dependence relationship and autocorrelation of situation sequences and establish mathematical prediction model [9]. It requests that situation sequences or their certain step difference satisfies the steady suppose, which is too strict to increase suitable scope. As one of GMs, GM(1,1) firstly weakens the randomness of situation sequences by using accumulation, secondly fits the born sequence through index curve, and then does regressive restitution after prediction, which can embody monotonously and slowly changing trend but hardly reflect some characteristics such as random rove and periodic fluctuation [10, 11]. Grey Verhulst is suitable to describe the situation sequences with swing development according to “S” or anti-“S” form [12], and the method dividing the changing line into several stages does not lack rationality, but the difficulty is how to predict the occurrence moment and lasting time of each stage [13]. RBFNN utilizes the nonlinear characteristic to describe the regulation contained in situation sequences [14]. However, evolving regulation of attack situation is infinite and changeable; a practical type neural network with small scale cannot solve well [15, 16].

Situation sequence contains massive complex and inconstant evolution trends, beyond the expression and prediction capability of traditional methods only by some formulas, functions or via some training [17, 18]. Most traditional methods suffer from the confliction among training samples, rely on data preprocessing and artificial intervention heavily, do not support incremental training, and need to rebuild model once situation sequence changes [19–21]. Therefore, a situation prediction method based on historical feature pattern extraction (HFPE) is presented. The method measures the similarity between historical feature from the aspects of pattern and accuracy and utilizes multiple order difference operation to discriminate trends. It searches similar indications from recorded historical situation sequence, measures the link intensity of occurred indication upon subsequent effect, and infers the recurrence possibilities of some effects according to current indication. An evolution algorithm is introduced to measure prediction deviation and improve the adaptability of prediction algorithm continuously via gradual fine-tuning.

This paper proceeds as follows: Section 2 discusses algorithm principle for HFPE.  Section 3 clarifies algorithm establishment and analysis.  Section 4 presents the experiment results and Section 5 concludes the paper.

## 2. Algorithm Principle

### 2.1. Basic Definition

Looking from mathematical form, the continuous time-varied curve, *z* = *f*(*t*), is commonly applied to describe the evolving process of attack situation. This curve is carried out by computer through sampling method, that is, to sample situation values with time interval *τ*, and then obtains discrete time sequences composed by (*t*_*k*_, *z*_*k*_), where *z*_*k*_ represents the situation value at moment *t*_*k*_. To facilitate the research, a basic definition is made as follows: let *G*(*i*, *m*) be the segmental subimage with *m* neighboring segments from moment *t*_*i*_, let *q*_*k*_ be the segmental gradient, let *Q*(*i*, *m*) be the gradient sequence, let (*q*_*i*_, *q*_*i*+1_,…, *q*_*i*+*m*−1_), *L*(*i*, *m*) be the characteristic spectrum of *Q*(*i*, *m*), let *O* be zero vector; then

(1) $$\begin{array}{c} q_{k}=\frac{z_{k+1}-z_{k}}{t_{k+1}-t_{k}},\\ L\left(i,m\right)=O,\;Q\left(i,m\right)=O,\\ L\left(i,m\right)=\frac{Q\left(i,m\right)}{||Q\left(i,m\right)||},\;Q\left(i,m\right)\neq O. \end{array}$$

For the *k*th component product of *L*(*i*, *m*), *l*_*i*+*k*_, the angle of inclination, *θ*_*i*+*k*_, can be defined as

(2) $$\begin{array}{c} \theta_{i+k}=\arctan l_{i+k},\;-\frac{\pi }{2}<\theta_{i+k}<\frac{\pi }{2}. \end{array}$$

The stretch rate from *Q*(*i*, *m*) to *Q*(*j*, *m*) can be calculated by *f*_*σ*_(*i*, *j*, *m*), which is defined as

(3) $$\begin{array}{c} f_{\sigma }\left(i,j,m\right)=1,\;Q\left(i,m\right)=O,\;\;Q\left(j,m\right)=O,\\ f_{\sigma }\left(i,j,m\right)=\frac{||Q\left(j,m\right)||}{||Q\left(i,m\right)||},\;Q\left(i,m\right)\neq O,\;\;Q\left(j,m\right)\neq O,\\ \text{not}\;\text{exist},\;\text{other}\;\text{conditions}. \end{array}$$

*γ*[*i*, *m*, *ρ*] is utilized to adjust the stretch rate, where *ρ* is the prediction steps.

The following three theorems through further analysis can be easily obtained: (1)  *L*(*i*, *m*) does not change with *G*(*i*, *m*); (2) if *L*(*i*, *m*) = *L*(*j*, *m*), then *Q*(*i*, *m*) is linear correlative with *Q*(*j*, *m*); (3) if *L*(*i*, *m*) = *L*(*j*, *m*), then *G*(*i*, *m*) can be the same with *G*(*j*, *m*) through translating and magnifying.

### 2.2. Prediction Principle

Looking from probability theory and statistics, similar situation curve shapes are more probably derived from similar origin, mechanism, and impact, subsequently resulting in a similar subsequent effect. From the point of view of statistics, when the precedence relations of sequences in time appear frequently, it usually meant that the logical causal relationship exists in a certain degree.

It is supposed that *G*(*i*, *m*) and *G*(*j*, *m*) are known historical feature subpatterns, from the same pattern, *t*_*i*_ < *t*_*j*_, and the further trend after *t* > *t*_*j*+*m*_ is unknown and needed to be predicted. If *G*(*i*, *m*) is similar with *G*(*j*, *m*), then it can be deduced that the origin, mechanism, and impact in [*t*_*j*_, *t*_*j*+*m*_) are similar with those in [*t*_*i*_, *t*_*i*+*m*_), and the history after *t*_*i*+*m*_ may be repeated after *t*_*j*+*m*_ with some differences. According to this principle, the slope of the line segment behind can be forecasted by

(4) $$\begin{array}{c} {\hat{q}}_{j+m+k}=f_{\sigma }\left(i,j,m\right)\times q_{i+m+k}. \end{array}$$

*ρ* is utilized to control the predicting steps. When *k* = 0,1, 2,…, *ρ* − 1, the trend prediction curve can be recurred by *τ* and ${\hat{q}}_{j+m+k}$.

### 2.3. Measurement System 

#### 2.3.1. Fitting Degree

Firstly calculate the angle cosine similarity between slope vectors, secondly introduce more order difference operators to obtain the trend difference of qualitative change and quantitative change, and then acquire the narrowing fitting degree by the difference of similarity degree and trend difference.

Let *ϕ*_*θ*_(*i*, *j*, *m*) represent the angle cosine similarity between *Q*(*i*, *m*) and *Q*(*j*, *m*); then

(5) ϕθ(i,j,m)=1, Q(i,m)=O, Q(j,m)=O,ϕθ(i,j,m)=Q(i,m)×QT(j,m)||Q(i,m)||×||Q(j,m)||,Q(i,m)≠O, Q(j,m)≠Oϕθ(i,j,m)=0, Q(i,m)=O, Q(j,m)≠Oor  Q(i,m)≠O, Q(j,m)=O.

The trend differences of qualitative change and quantitative change are denoted by *ϕ*_1_(*x*) and *ϕ*_2_(*x*), respectively, and the former of which stands for the pattern difference, and the latter stands for the accuracy difference. The above two parameters can be derived by

(6) ϕ1(x)={+1,x>00,x=0−1,x<0,ϕ2(x)=sinx.

Thus the composite trend, *ϕ*_⊥_(*x*), can be defined by

(7) $$\begin{array}{c} \phi_{⊥}\left(x\right)=0.2\times \phi_{1}\left(x\right)+0.8\times \phi_{2}\left(x\right). \end{array}$$

Let ∇ represent backward difference operator, and define

(8) $$\begin{array}{c} \nabla^{0}\theta_{k}=\theta_{k}, \end{array}$$

and then the *α* order differential recursive equation can be obtained by

(9) $$\begin{array}{c} \nabla^{\alpha }\theta_{k}=0.5\times \left(\nabla^{\alpha -1}\theta_{k}-\nabla^{\alpha -1}\theta_{k-1}\right), \end{array}$$

in which *α* is a positive integer, and (9) meets

(10) $$\begin{array}{c} \frac{\pi }{2}<\nabla^{\alpha }\theta_{k}<\frac{\pi }{2}. \end{array}$$

Let *ϕ*_∇_(*i*, *j*, *m*) denote the trend difference between the feature patterns *L*(*i*, *m*) and *L*(*j*, *m*); then

(11) $$\begin{array}{c} \phi_{\nabla }\left(i,j,m\right)=\frac{2\sum_{\alpha =0}^{m-1}\sum_{k=\alpha }^{m-1}\left|\phi_{⊥}\left(\nabla^{\alpha }\theta_{i+k}\right)-\phi_{⊥}\left(\nabla^{\alpha }\theta_{j+k}\right)\right|}{m\left(m+1\right)}. \end{array}$$

The fitting degree function, *ϕ*(*i*, *j*, *m*), can be defined by

(12) $$\begin{array}{c} \phi \left(i,j,m\right)=\phi_{\theta }\left(i,j,m\right)-\phi_{\nabla }\left(i,j,m\right), \end{array}$$

where the large value of *ϕ*(*i*, *j*, *m*) represents a fine fitting, and for −1 < *ϕ*_*θ*_(*i*, *j*, *m*) ≤ 1 and 0 < *ϕ*_*θ*_(*i*, *j*, *m*) ≤ 2, it can be derived that

(13) $$\begin{array}{c} -3<\phi_{\theta }\left(i,j,m\right)\leq 1. \end{array}$$

The occurrence probability of *ϕ*_*θ*_(*i*, *j*, *m*) > 0 may be 50% statistically, which is too big. Therefore, it is necessary to subtract the penalty term, *ϕ*_∇_(*i*, *j*, *m*), and filter *ϕ*(*i*, *j*, *m*) by the threshold *ε*_*ϕ*_(0 < *ε*_*ϕ*_ < 1) to narrow the fitting degree.

#### 2.3.2. Universality Degree

Divide the attack situation subsequence into two parts, that is, occurred indication and subsequent effect; the values of the domination intensity of the former to the latter (or call link intensity between the two parts) may be high or low, some of which have a far-ranging representative, and some just have rare earth especially instance. If all the values are treated evenly, then the prediction accuracy will be affected seriously, so it is important to outstand inevitable link of the high intensity and weaken accidental link of the low intensity.

Let *χ*[*k*, *m*, *ρ*] be the universality value of *Q*(*k*, *m* + *ρ*) in the historical feature pattern *G*(0, *n*), where *χ*_max⁡_ can be derived by

(14) $$\begin{array}{c} \chi_{\max}=\max\left\{\chi \left[k,m,\rho \right]\;|\;0\leq k<n-m\right\}. \end{array}$$

The value of *χ*_max⁡_ will be updated with the change of *χ*[*k*, *m*, *ρ*] and can be accessed directly without waiting to calculate.

The universality value can be shined upon to universality degree in (0, *n* − *m*] by function *f*_*χ*_(*k*, *m*, *ρ*), which is shown as follows:

(15) fχ(k,m,ρ)=(n−m) ×(1+2π−1arctan(χ[k,m,ρ]−χmax⁡)).

The larger value of universality degree reflects finer representativeness of *Q*(*k*, *m*) and its extension and more exact patterns predicted by *Q*(*k*, *m* + *ρ*). Otherwise, *Q*(*k*, *m* + *ρ*) is just a special example, and the prediction effect is worse.

#### 2.3.3. Contrast Degree

The predication results of situation are usually impacted by link intensity of several different weights. The function mechanism often changes; that is, sometimes they work with a community decision and sometimes with an individual domination. Therefore, it is necessary to trace and adjust between outstanding statistics effect and showing individual advantage.

It is supposed that ${\tilde{w}}_{1},{\tilde{w}}_{2},\ldots ,{\tilde{w}}_{n}$ are not normalized weights, which can be adjusted to ${\tilde{w}}_{1}^{\eta },{\tilde{w}}_{2}^{\eta },\ldots ,{\tilde{w}}_{n}^{\eta }$ by sensitization index *η* (*η* > 0). Then the standardized weight *w*_*k*_ can be derived by

(16) $$\begin{array}{c} w_{k}=\frac{{\tilde{w}}_{k}^{\eta }}{\sum_{k=1}^{n}{\tilde{w}}_{k}^{\eta }}, \end{array}$$

and comparison degree *w*_*i*_/*w*_*j*_ can be obtained by

(17) $$\begin{array}{c} \frac{w_{i}}{w_{j}}=\frac{{\tilde{w}}_{i}^{\eta }}{{\tilde{w}}_{j}^{\eta }}. \end{array}$$

If ${\tilde{w}}_{i}\neq {\tilde{w}}_{j}$, we can suppose ${\tilde{w}}_{i}<{\tilde{w}}_{j}$; then five generalized cutoff points can be acquired; that is, $0<{\tilde{w}}_{i}/{\tilde{w}}_{j}<1<{\tilde{w}}_{j}/{\tilde{w}}_{i}<\infty$.

Equation (16) can be derived into the form of ${\tilde{w}}_{k}^{\eta }=w_{k}\times \xi$ by

(18) $$\begin{array}{c} \frac{{\tilde{w}}_{k}^{\eta \times x}}{\sum_{k=1}^{n}{\tilde{w}}_{k}^{\eta \times x}}=\frac{{\left(w_{k}\times \xi \right)}^{x}}{\sum_{k=1}^{n}{\left(w_{k}\times \xi \right)}^{x}}=\frac{w_{k}^{x}}{\sum_{k=1}^{n}w_{k}^{x}}, \end{array}$$

where *η* is utilized to adjust comparison degree; that is, 0 < *η* < 1 outstands statistics effect, *η* > 1 shows individual advantage, and *η* = 1 maintains the present status.

## 3. Algorithm Establishment and Analysis

### 3.1. Prediction Algorithm

**Figure 1:**
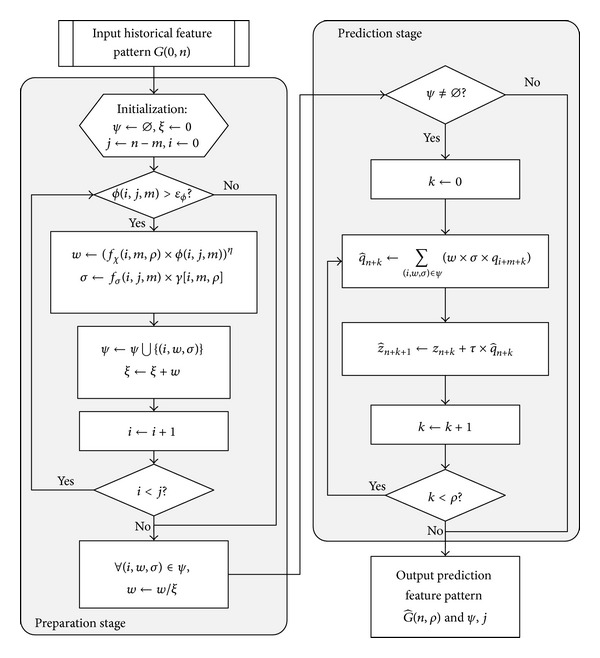
Prediction algorithm flow chart.

The prediction algorithm flow is given in Figure 1. According to historical feature pattern *G*(0, *n* − 1) and occurred indication *G*(*n* − *m*, *m*), the algorithm can predict subsequent effect $\hat{G}(n,\rho )$ through two main stages, that is, preparation stage and prediction stage, which are marked in the chart.

As shown in the figure, the preparation part circularly promotes the sliding window *G*(*i*, *m*), selects poor values of fitting degree *Q*(*i*, *m*) to reject, and sensitizes the product of universality degree *f*_*χ*_(*i*, *m*, *ρ*) and fitting degree *ϕ*(*i*, *j*, *m*), which is assigned to *w*. The prediction part first checks whether historical feature pattern set *ψ* has record. What calls for special attentions is that the value of *Q*(*i*, *m*) in the sliding window or the fitting degree value of it with *Q*(*j*, *m*) in the occurred indication cannot be too small, because the smaller the above value, the poorer the contribution to prediction value ${\hat{q}}_{n+k}$.

### 3.2. Evolution Algorithm

Evolution algorithm is introduced to measure predicting deviation from the views of pattern and accuracy, which can be fine-tuned to raise the adaptability of prediction algorithm.

The accuracy of adjusting *η* to *η* × *x* can be derived by

(19) $$\begin{array}{c} f_{k}\left(x\right)=\frac{\sum_{(i,w,\sigma )\in \psi }^{}\left(w^{x}\times \phi \left(i+m,n,\rho \right)\right)}{\sum_{(i,w,\sigma )\in \psi }^{}w^{x}}, \end{array}$$

which is based on current weight set and (18) and meets −3 < *f*_*k*_(*x*) ≤ 1. And the definition can be popularized to *f*_Λ_(*x*_1_, *x*_2_,…), only when *f*_*k*_(*x*_*i*_) is the largest value first met in {*f*_*k*_(*x*_1_), *f*_*k*_(*x*_2_),…}, which is in ascending order by *k* of *x*_*k*_; it can be obtained that

(20) $$\begin{array}{c} f_{\Lambda }\left(x_{1},x_{2},\ldots \right)=x_{i}. \end{array}$$

**Figure 2:**
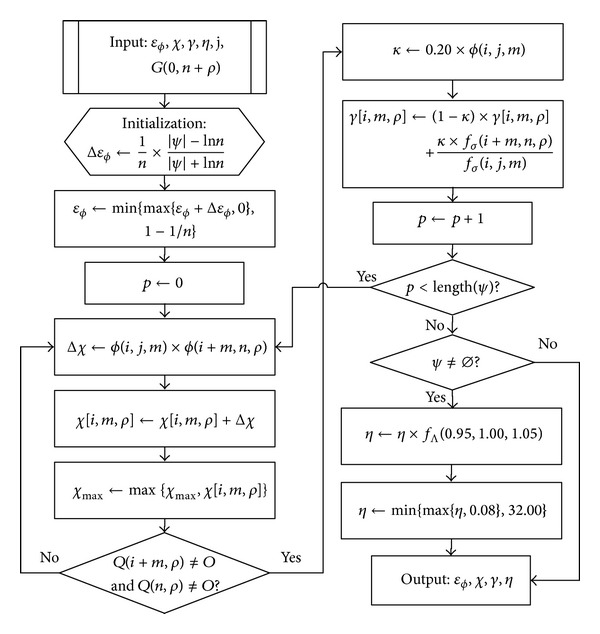
Evolution algorithm flow chart.

As shown in Figure 2, the evolution algorithm carries on the variables and results of the prediction algorithm and works to promote adaptability after acquiring measured value. Δ*ε*_*ϕ*_ is an adjustment variable for *ε*_*ϕ*_ and meets −*n* ^−1^ ≤ Δ*ε*_*ϕ*_ ≤ *n* ^−1^. If *n* rises or the distance between |*ψ*| and ln⁡*n* drops, then the adjustment amplitude becomes lower, else becomes higher. If |*ψ*| < ln⁡*n*, then decrease the threshold to soften the terms, else increase the threshold. Δ*χ* is calculated through fitting degree, *ϕ*(*i*, *j*, *m*), selected by the prediction algorithm, and meeting 0 < *ϕ*(*i*, *j*, *m*) ≤ 1. When the fitting of *Q*(*i*, *m*) and *Q*(*j*, *m*) is poor, the adjustment to *χ*[*i*, *m*, *ρ*] needs to be cautious; that is, if *ϕ*(*i* + *m*, *n*, *ρ*) > 0, then it needs to raise the value of *χ*[*i*, *m*, *ρ*], and if the extension value *Q*(*i* + *m*, *ρ*) approximates *Q*(*n*, *ρ*), then the prediction according to *Q*(*i* + *m*, *ρ*) is accurate, and the value raising can be large. To determine *η*, select the best one among value lowering by 5%, current value, and value rising by 5%, and restrict it by a reasonable range to prevent passivating or sharpening.

### 3.3. Example Analysis

**Figure 3:**
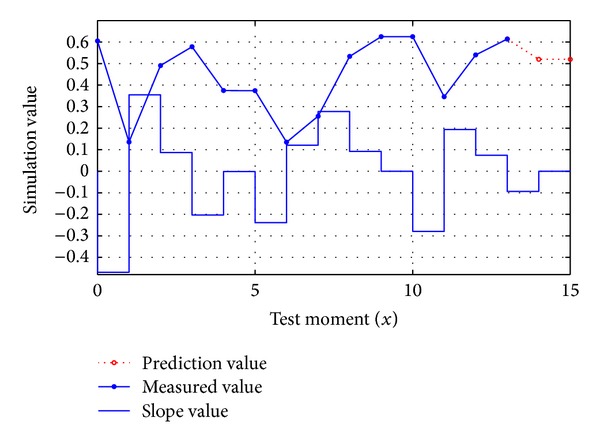
Trend prediction illustration.

Figure 3 gives an example of predicting $\hat{G}(13,2)$ according to the historical feature pattern *G*(0,13), in which the value of (*n*, *m*, *ρ*) is (13,3, 2), *ε*_*ϕ*_ = 0.2, *χ*[*i*, *m*, *ρ*] = 0.0, *χ*_max⁡_ = 0.0, *γ*[*i*, *m*, *ρ*] = 1.0, and *η* = 1.0.

According to the prediction algorithm, it can be known through comparing all the values of *Q*(*i*, 3) with *Q*(10,3) that when *i* = 0 and *ϕ*(0,10,3) = 1.00, so *Q*(0,3) is selected, and when *i* = 5 and *ϕ*(5,10,3) = 0.42, so *Q*(5,3)is also selected, and other values are excluded for they meet *ϕ*(*i*, 10,3) ≤ *ε*_*ϕ*_, which are partly listed in Table 1. When *i* = 5, the slope becomes larger at *t* = 7 and smaller at *t* = 12, which are reflected through ∇^1^ *θ*_7_ > 0 and ∇^1^ *θ*_12_ < 0 derived by (9), and the relative penalty value is recorded by *ϕ*_∇_(5,10,3). And through normalization process, the elements of set *ψ* are (0,0.705,0.67) and (5,0.295,0.98). Thus, the prediction value ${\hat{q}}_{13}$ is equal to 0.705 × 0.67 × (−1.29) + 0.295 × 0.98 × 0.50, and so forth, and *ρ* step trend can be predicted. It can be seen that this scheme has the ability to identify multiple long-range correlation contained in the same situation sequence.

This part is analyzed according to evolution algorithm. Assuming that *q*_13_ = −0.86 and *q*_14_ = 0.00, so |*ψ*| = 2, which is smaller than ln⁡13; thus, the value of *ε*_*ϕ*_ needs to be lower, and once |*ψ*| > 2, then raise the value of *ε*_*ϕ*_. It can be known that the changing value of universality degree *χ*[0,3, 2] is 1.00 × 1.00, and raising this degree can strengthen the role of vector *Q*(0,5). The changing value of *χ*[5,3, 2] is 0.42 × (−1.85), and lowering this value can weaken the interference of vector *Q*(5,5). To bridge the gap between epitaxial scale and measured scale, *γ*[0,3, 2] is adjusted to (1 − 0.20) × 1.00 + 0.20 × 0.67/0.67, and *γ*[5,3, 2] is adjusted to (1 − 0.08) × 1.00 + 0.08 × 1.72/0.98. And for (*f*_*κ*_(0.95), *f*_*κ*_(1.00), *f*_*κ*_(1.05)) = (0.13,0.16,0.19), the value of *η* needs to rise.  Table 2 shows that *f*_*χ*_(5,3, 2) becomes smaller, and *η* becomes larger with continued evolution, which results in rapid rise of *w*_0_/*w*_5_, and approach between prediction value and measured value.

With the passage of time, *m* and *ρ* keep unchanged, *n* grows linearly, and the algorithm can delete stale data, save recent data, and correct fitting threshold and universality degree. The above process can be complicated not only by autonomous evolution, but also by artificially modified parameters.

## 4. Experiment Results Analysis

The traditional indexes utilized to measure the prediction accuracy include mean absolute error (MAE), standard deviation error (SDE), and mean absolute percentage error (MAPE) [21] derived by

(21) $$\begin{array}{c} \text{MAPE}=\frac{1}{\rho }\overset{n+\rho }{\underset{k=n+1}{\sum }}\left|\frac{{\hat{z}}_{k}-z_{k}}{z_{k}}\right|. \end{array}$$

This section selects MAPE to obtain the relative error between prediction pattern and measured pattern, which is denoted by *E*_*r*_. The standard deviation of *ρ* relative error components is denoted by *E*_std_.

### 4.1. Experiment  1

**Figure 4:**
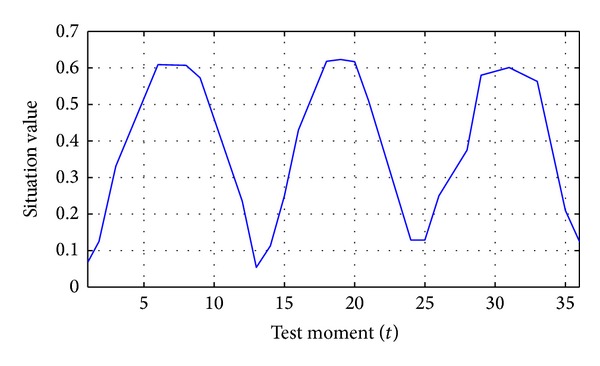
Historical records of network attack situation.

Figure 4 is a critical subsequence selected from actual network attack situation records, which includes various features such as ascent trend, saturation trend, decline trend, periodic fluctuation, and stochastic disturbance.

**Figure 5:**
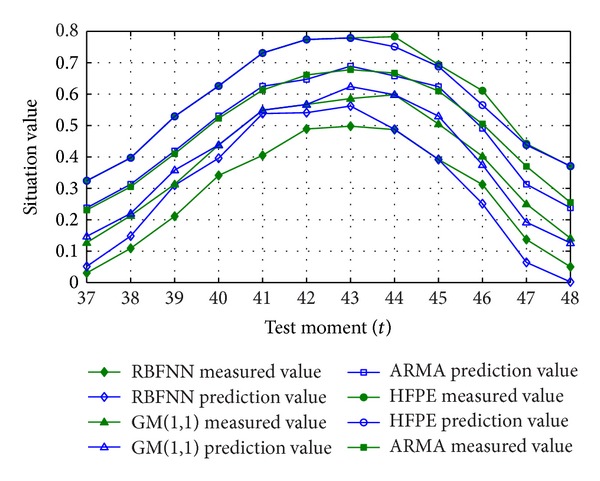
Prediction results of network attack situation.

From the view of the experimental prediction results, the relative errors of HFPE, ARMA, GM(1,1), and RBFNN are 3.28%, 5.89%, 7.18%, and 16.11%, respectively. As shown in Figure 5, in the experiment, ARMA, GM(1,1), and RBFNN need to be artificially identified and protected against cyclical situation fluctuations. The difference transformation utilizes 12 as the distance and is restored after prediction to prevent poor prediction effect; otherwise, the relative errors of GM(1,1) and RBFNN may reach 59.67% and 73.99%, respectively. However, the above method is special, cannot be spread for that data preprocessing of these algorithms does not exist in universal law. On the contrary, HFPE can maintain adaptation to complicated and changeable trends but does not need data preprocessing or artificial cognition.

### 4.2. Experiment  2

This experiment is to randomly choose subsequences with similar parts, repeat 20 times, and then calculate the average value.

**Figure 6:**
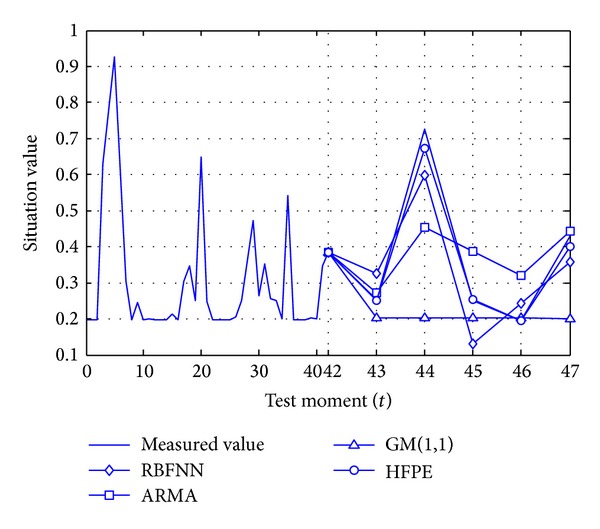
Prediction results of network attack situation.

From the view of the experimental prediction results, the relative errors of HFPE, ARMA, GM(1,1), and RBFNN are 8.09%, 20.89%, 44.89%, and 34.75%, respectively. If the situation sequence selected does not exist in any principle, then the relative errors will be 3.96%, 21.72%, 37.47%, and 53.54%, respectively.  Figure 6 shows one group of data, in which *t* = 42 is a boundary for historical feature pattern and prediction feature pattern.

If put all groups of the historical feature patterns into a new long sequence, and repeat above prediction, then the performance of ARMA and GM(1,1) drops rapidly, and that of HFPE does not change much for that longer sequence containing more correlation is benefit to prediction.

### 4.3. Experiment  3

To compare differences among four algorithms, random data are utilized to simulate situation sequences. First, extract random data with *ξ* bits from the entropy pool of Windows 7 system. Then randomly gather subsequence with 16 bits, the former 8 bits of which are occurred indication and the latter 8 bits are subsequent effect. Thirdly, splice occurred indication behind the random sequence to form a historical feature pattern and treat the subsequent effect as a prediction feature pattern. Let us make 100 groups of experiments to test each algorithm's capacity in resisting random interference and in identifying the correlation with far distance. The average results are listed in Table 3.

It can be found from the table data that HFPE has the best performance among the four algorithms. When the scale of experiment is large, this conclusion can be repeated well. And ARMA and RBFNN cannot deal with the random sequences with long bits, while HFPE can perform smoothly.

## 5. Conclusion

This paper proposes a prediction method based on historical feature pattern, that is, HFPE. The main principle of this algorithm is shown as follows. Fitting degree is introduced to measure the similarity among subsequences from the views of pattern and accuracy. Universality degree is utilized to test the representation of subsequence and its epitaxy. Contrast of the weight system is adjusted by sensitized index, which gives prominence to statistical effect in passivation area and highlights individual strengths in sharpening area. Prediction algorithm and evolution algorithm are proposed to predict situation results according to historical feature patterns. HFPE algorithm maximally reserves the rules in the situation sequences, does not need data preprocessing, and can adapt to the situation changes automatically. The experiment results prove the performance of HFPE.

## Figures and Tables

**Table 1:** Similarity metric and punishment value.

*i*	0	2	3	5	6	8
*ϕ*_Θ_	1.00	−0.85	0.42	0.72	0.32	−0.32
*ϕ*_∇_	0.00	1.01	0.34	0.30	0.30	0.68

**Table 2:** Effects of prediction and evolution.

	1	3	5	7	9
*ω*_0_	0.71	0.95	0.98	0.99	1.00
*ω*_5_	0.29	0.05	0.02	0.01	0.00
${\hat{q}}_{13}$	−0.46	−0.78	−0.83	−0.85	−0.86
${\hat{q}}_{14}$	0.00	0.00	0.00	0.00	0.00
*ε*_*ϕ*_	0.19	0.17	0.15	0.13	0.11
*f*_*χ*_(0,3, 2)	10.00	10.00	10.00	10.00	10.00
*f*_*χ*_(5,3, 2)	3.27	1.18	0.72	0.51	0.40
*γ*[0,3, 2]	1.00	1.00	1.00	1.00	1.00
*γ*[5,3, 2]	1.06	1.17	1.27	1.35	1.41
*η*	1.05	1.16	1.28	1.41	1.55

**Table 3:** Prediction effects of network attack situation.

	*ξ*	*ε*_*ϕ*_	*E*_*r*_	*E*_std_
HFPE	1.2 × 10^2^	0.930	2.49	0.09
	1.2 × 10^3^	0.980	1.75	0.09
	1.0 × 10^6^	0.998	1.88	0.09

ARMA	1.2 × 10^2^	—	32.96	0.15

GM(1,1)	1.2 × 10^2^	—	49.32	0.20
	1.2 × 10^3^	—	51.06	0.18

RBFNN	1.2 × 10^2^	—	27.87	0.23
